# Serum amyloid A levels and alpha 2 and gamma globulins on serum protein electrophoresis in cats exposed to and infected with *Leishmania infantum*

**DOI:** 10.1186/s13071-021-04710-9

**Published:** 2021-04-21

**Authors:** Giulia Savioli, Joy Archer, Emanuele Brianti, Giovanni Benelli, Manuela Schnyder, Roberta Iatta, Domenico Otranto, Cinzia Cantacessi

**Affiliations:** 1 Department of Veterinary Medicine, University of Cambridge, Cambridge, UK; 2 Dipartimento di Scienze Veterinarie, Università degli Studi di Messina, Messina, Italy; 3 Department of Agriculture, Food and Environment, University of Pisa, Pisa, Italy; 4 Institute of Parasitology, Vetsuisse Faculty, University of Zurich, Zurich, Switzerland; 5 Dipartimento di Medicina Veterinaria, Università degli Studi di Bari Aldo Moro, Bari, Italy

**Keywords:** Acute phase protein, Alpha 2 globulins, Feline leishmaniosis, Gamma globulins, *Leishmania infantum*, Phlebotomine sand flies, Serum amyloid A, Serum protein electrophoresis

## Abstract

**Background:**

Dogs are the main reservoir hosts of *Leishmania infantum*; nevertheless, recent investigations indicate a likely role for cats in the epidemiology of *Leishmania* infection. Feline leishmaniosis (FeL) remains poorly characterised, partly due to the lack of suitable diagnostic tools. This study aimed to compare serum amyloid A (SAA) levels and serum protein electrophoresis (SPE) profiles (specifically, alpha 2 and gamma globulins) in cats naturally exposed to or infected by *L. infantum* from southern Italy *versus* those of healthy controls and *versus* cats with neoplastic or inflammatory conditions from non-endemic areas.

**Methods:**

Serum or plasma samples from four cohorts of cats were analysed for SAA levels and by SPE: (i) G1: healthy controls from *Leishmania-*non-endemic regions of Switzerland; (ii) G2: cats pre-diagnosed with neoplastic or inflammatory conditions available from the University of Cambridge sample archive; (iii) G3: *L. infantum*-seropositive, quantitative (q)PCR-negative cats from southern Italy; (iv) G4: *L. infantum*-seropositive and qPCR-positive cats from southern Italy. SAA data were assessed for normality and homoscedasticity using the Shapiro–Wilk and Levene’s tests, respectively; the Kruskall–Wallis test, followed by Dunn’s test with Bonferroni correction were subsequently used to compare SAA serum levels between groups. A weighted generalised linear model with a binomial distribution was used to assess statistically significant differences in the numbers of animals displaying elevated gamma globulins and increased alpha 2 globulins between groups.

**Results:**

Overall, 68 samples were analysed (G1: *n* = 16, G2: *n* = 20, G3: *n* = 20, G4: *n* = 12). Cats suffering from neoplastic and inflammatory conditions (G2 ) showed significantly higher SAA levels than healthy controls (G1) (median values [interquartile range]: G1: 0.00 [0.00–0.00] mg/l* versus* G2: 0.85 [0.00–49.55] mg/l). G2, G3 and G4 cats showed higher percentages of individuals with increased alpha 2 globulins (percentages ± standard error: G1 = 20.0% ± 10.3, G2 = 80.0% ± 8.9, G3 = 70.0% ± 10.2, G4 = 75.0% ± 12.5) and gamma globulins (G1 = 0.0% ± 0, G2 = 65.0% ± 10.7, G3 = 50.0% ± 11.2, G4 = 58.3% ± 14.2) than healthy control cats (G1). For all three markers, no significant difference between cats within G2, G3 and G4 was recorded.

**Conclusions:**

This study indicates that the proportions of animals with elevated levels of alpha 2 and gamma globulins are significantly higher in cats exposed to and infected with *L. infantum*. Levels of SAA and alpha 2 and gamma globulins may not be used to differentiate between *L. infantum* infection or exposure, and neoplastic and/or inflammatory conditions.

**Graphic Abstract:**

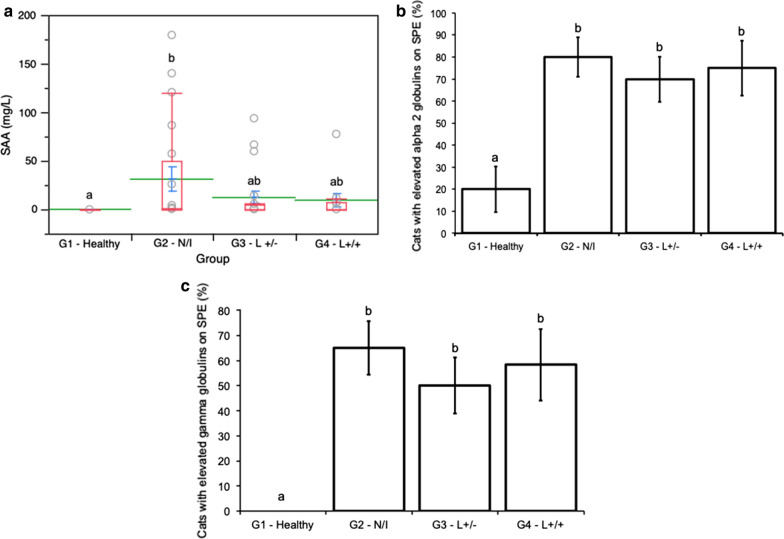

## Background

Zoonotic visceral leishmaniosis (ZVL) is a neglected disease caused by *Leishmania infantum*, transmitted by *Phlebotomus* spp. sand flies in the Old World [1]. The disease is distributed worldwide and endemic in many regions, including the Mediterranean basin [2]. Dogs are the main reservoir hosts of *L. infantum* [3]. Canine leishmaniosis (CanL) is endemic in Italy, and the prevalence of infection has increased with its spread from hyper-endemic southern and central areas towards northern regions [4]. The epidemiological role of other animal species as alternative reservoir hosts of *L. infantum* has long been overlooked. Nevertheless, recent epidemiological investigations in other species have pointed towards the likely implication of  domestic and wild felids in parasite circulation [5–8]. In particular, endemic foci of feline leishmaniosis (FeL) have recently been described in southern Italy, and the overall prevalence of feline infection in these areas is likely underestimated [9, 10].

FeL is often subclinical [11–13], with clinical cases mainly characterised by cutaneous lesions, with crusty or nodular dermatitis being the most common presentation [14], whilst lymphadenomegaly seldom reported [5]. Typical diagnostic samples include skin (lesions), lymph node, bone marrow and blood [14, 15]. When present, clinical signs are non-specific, and alterations in haematological, biochemical and urinary profiles, typically detected in dogs with leishmaniosis [16], may support suspicion of FeL. Due to the frequently subclinical nature of the infection, a straightforward measure of disease progression and response to treatment would facilitate the clinical management of FeL. The measurement of acute phase proteins (APPs), including C-reactive protein (CRP), haptoglobin and serum amyloid A (SAA), is increasingly being applied to the diagnosis, monitoring and prognosis of a range of veterinary inflammatory conditions [17, 18], including *L. infantum* infection in dogs [16, 19].

The acute phase response is an early, non-specific defence mechanism characterised by the release of proinflammatory cytokines that stimulate an increase in serum APPs in response to infection, inflammation, tissue injury, neoplasia and other processes [20]. In dogs naturally infected by *L. infantum*, a significant increase in the release of APPs, including CRP, haptoglobin and ceruloplasmin is observed, irrespective of the occurrence of clinical signs [19]. SAA is considered to be a less sensitive indicator of CanL compared to other APPs [21]; nevertheless, SAA is a major indicator of feline inflammatory conditions, including trauma, post-operative inflammation and sepsis [22–24]. SAA is substantially increased in cats with clinical signs of vector-borne infections, such as those caused by *Hepatozoon felis* and *Babesia vogeli*, compared to uninfected and subclinically infected cats [25]. Recently, protein-related laboratory abnormalities were reported in tigers naturally infected by *L. infantum* [26]. While information on serum APP levels in cats infected with vector-borne pathogens remains limited [25], these findings may suggest a useful diagnostic and prognostic role for SAA.

Serum protein electrophoresis (SPE) is a straightforward method to measure specific serum proteins and separate these into fractions. The alpha 2 fraction in SPE contains a number of APPs; thus its quantification may be useful to diagnose inflammatory processes [27]. However, the levels of alpha 2 globulin in SPE in FeL have, thus far, not been investigated in detail. In one study, FeL-infected cats showed significantly lower levels of the alpha 2 globulin fraction compared to uninfected cats, with median alpha 2 globulin levels remaining within the reference range for infected cats [28]. In another study, FeL-positive cats did not show significantly higher alpha 2 globulin levels compared to negative controls [29]. SPE is commonly used to identify and characterise gammopathy in dogs infected by *L. infantum* [16, 30]. Gammopathy normally occurs in both CanL and FeL [15, 28, 29].

In this scenario, the aim of the present study was to evaluate and compare the serum SAA levels and SPE profiles, specifically those of alpha 2 and gamma globulins, in cats from a ZVL-endemic area naturally exposed to or infected by *L. infantum* *versus* healthy cats and cats with pre-diagnosed neoplastic or inflammatory conditions.

## Methods

### Enrolment and diagnostic procedures

A total of 68 serum or plasma samples were obtained from four groups of cats:

- Group 1 (G1): 16 healthy control cats from *Leishmania-*non-endemic regions of Switzerland (north of the Alps). The samples originated from the archives of the Clinical Laboratory or the Institute of Parasitology, Vetsuisse Faculty, University of Zurich (VetLab Zurich and IPZ, respectively), and included healthy stray cats recruited into a spay/neuter programme (*n* = 5), healthy blood donors (*n* = 6) and healthy laboratory cats (*n* = 5). All cats were recorded as clinically healthy following a thorough clinical examination by an accredited veterinary surgeon. Routine haematology and biochemistry analyses were performed with samples from blood donors, and animals that had travelled abroad were excluded. Cats from the spay and neuter programme were recruited only from the north of the Alps, a *Leishmania*-free area. Laboratory animals are born and kept in controlled experimental units, with no exposure to Phlebotominae. The G1 group included 3 plasma and 13 serum samples.
- Group 2 (G2): 20 cats pre-diagnosed with neoplastic or inflammatory conditions selected from the sample archive of the Queen’s Veterinary School Hospital of the University of Cambridge. These cats originated from a *Leishmania*-free, namely the UK, and had not travelled abroad.
- Group 3 (G3): 20 cats from southern Italy (i.e. Aeolian Islands, Sicily and Apulia regions) testing seropositive for *L. infantum* by immune-fluorescence antibody test (IFAT), but negative by quantitative (real-time) PCR (qPCR).
- Group 4 (G4): 12 cats from southern Italy (Aeolian Islands, Sicily) testing seropositive by IFAT and qPCR positive for *L. infantum*.

G3 and G4 samples were derived from previous published studies on FeL conducted in southern Italy [10]. In particular, all cats included in the study by Otranto et al. [10] were tested for co-infections with other vector-borne diseases (VBDs) (i.e. *Anaplasma* spp., *Babesia* spp., *Ehrlichia* spp., *Hepatozoon* spp. and *Bartonella* spp.) as well as with feline leukaemia and feline immunodeficiency viruses (FeLV and FIV, respectively). Only samples from cats that tested positive for* Leishmania*, but negative for other VBDs and FIV and FeLV infections, were assessed in the present study.

Detection of antibodies against *L. infantum* was performed by IFAT, as described by Otranto et al. [31], using a cut-off value of 1:80 [32]. Blood and conjunctival swabs from the enrolled cats were subjected to DNA extraction, using the DNeasy Blood & Tissue Extraction Kit (Qiagen, Hilden, Germany), according to the manufacturer’s instructions. The detection of a *L. infantum* kinetoplast DNA minicircle fragment (120 bp) was achieved by qPCR, using primers, probes and protocol described elsewhere [33].

### SAA and serum protein electrophoresis

The SAA concentrations were determined using a turbidimetric immunoassay kit (LZ-SAA; Eiken Chemical Co., Tokyo, Japan), with analysis on an automated analyser (Olympus AU480; Beckman Coulter Inc., Fullerton, USA). SPE was performed on alkaline buffered agarose gels using the Hydragel Protein(e) K20 electrophoresis system (SEBIA, Lisses, France), according to the manufacturer’s instructions, using 10-µl of each serum sample. The gels were analysed with the Perfection V700 Photo imaging densitometer (Seiko Epson Corp., Suwa, Nagano, Japan), using SEBIA Phoresis Rel 8.6.3 software (Magiras Diagnostics, Likovrisi, Greece). Total protein was measured using the biuret method on an automated analyser (Olympus AU480; Beckman Coulter Inc.). The numbers of cats whose plasma/sera displayed increased alpha 2 and gamma peaks on SPE were counted for each group according to reference intervals calculated by Taylor and colleagues [27]. Clonality of increased gamma peaks was assessed, with monoclonal gammopathies (as opposed to polyclonal gammopathies) defined as showing a narrow gamma peak, with a base width similar to the albumin peak or with peak height exceeding four-fold the peak width [27].

### Statistical analyses

The values of SAA within each group were neither normally distributed (Shapiro–Wilk test, *W* = 0.47, *P* < 0.001) nor homoscedastic (Levene’s test, *F*_3, 63_ = 11.30, *P* < 0.001). Data transformation did not allow distribution normalisation nor variance homogenisation. Therefore, non-parametric statistical tests were used. The Kruskall–Wallis test, followed by Dunn’s multiple comparison test with Bonferroni correction, was used to compare SAA serum levels between groups. Standard errors for the proportions (SEp) were calculated as: ${\text{SEp}} = \sqrt {p\left( {1 - p} \right)/n}$.

Differences in the numbers of cats displaying increased alpha 2 globulins and gamma globulins between groups were analysed by using a weighted generalised linear model (GLZ) with a binomial distribution to model increased alpha 2 globulins and gamma globulins outcomes. For both parameters, a GLZ with a fixed factor was used: *y* = *Xß* + *ε*, where* y* is the vector of the observation (i.e. outcome: increased = 1, not increased = 0), *X* is the incidence matrix, *ß* is the vector of the fixed effect (i.e. the tested group) and* ε* is the vector of the random residual effects (*P* = 0.05).

All statistical analyses were performed with JMP® 9 (SAS Institute, Cary, NC, USA) with *α* = 0.05 as threshold to detect significant differences.

## Results

Of 68 samples analysed, one G1 (healthy control) sample did not undergo SPE, while one G4 (positive for *L. infantum* by both IFAT and qPCR) sample did not undergo SAA analysis as insufficient sample was available (Table 1).

**Table 1:** Number of sampled cats, and serum protein electrophoresis and serum amyloid A results

Variable	GROUP 1 Healthy control	GROUP 2 Neoplastic or inflammatory conditions	GROUP 3 *Leishmania infantum* seropositive, qPCR-negative	GROUP 4 *L. infantum* seropositive, qPCR positive
Total number of samples analysed	16 (15 for SPE)	20	20	12 (11 for SAA)
SAA results (mg/l)^a^	0.00 (0.00–0.00)	0.85 (0.00–49.55)	0.00 (0.00–4.53)	0.00 (0.00–7.5)
SPE results^b^				
Alpha 2 globulins increased	3 (20.0 ± 10.3)	16 (80.0 ± 8.9)	14 (70.0 ± 10.2)	9 (75.0 ± 12.5)
Gamma globulins increased	0 (0 ± 0)	13 (65.0 ± 10.7)	10 (50.0 ± 11.2)	7 (58.3 ± 14.2)

*qPCR* Quantitative (real-time) PCR,* SAA* serum amyloid A,* SPE* serum protein electrophoresis

Reference ranges: SAA (mg/l): < 0.5; alpha 2 globulins (g/l) [27]: 2.94–10.25; gamma globulins (g/l) [27]): 4.33–21.40

^a^Data presented as median (interquartile range)

^b^Data presented as a number (percentage ± standard error on the percentage [SEP])

**Fig. 1:**
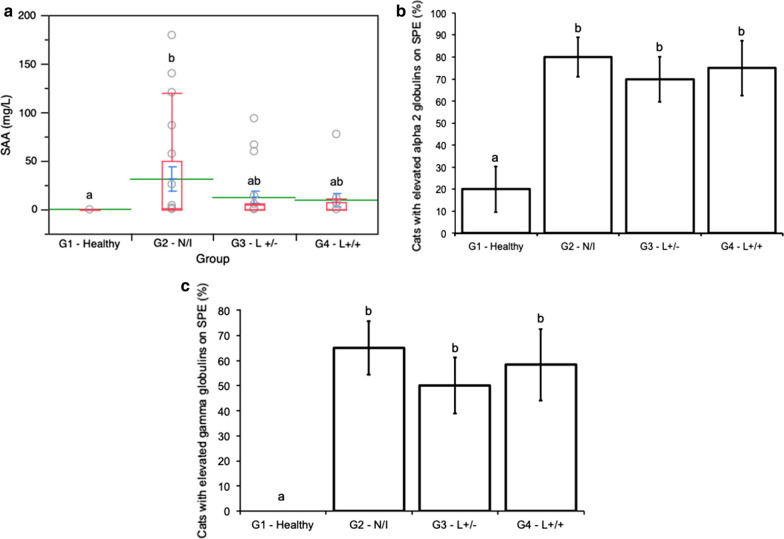
Serum amyloid A (*SAA*) concentrations (**a**), and proportions of cats showing increased alpha 2 globulins (**b**) and increased gamma globulins (**c**) on serum protein electrophoresis (*SPE*), in healthy control cats (*G1*: *n* = 16; 15 serum samples for SPE), cats affected by neoplastic or inflammatory conditions  (*N/I*) (*G2*: *n* = 20), cats seropositive and qPCR-negative for *Leishmania infantum* (*L+/-*) (*G3*: *n* = 20) and cats seropositive and qPCR-positive for *L. infantum* (*L+/+*) (*G4*: *n* = 12; 11 serum samples for SAA). **a** Red boxplots indicate the median (solid line) within each box and the range of dispersion (lower and upper quartiles and outliers); means and standard errors are represented by green lines and blue T-bars, respectively; the letters above boxplots indicate significant differences among groups (Dunn’s test with Bonferroni correction, *P* < 0.05). **b**, **c** letters above columns indicate significant differences among groups (generalised linear model, binomial distribution, *P* < 0.05)

Median values and interquartile ranges of SAA concentration in pathological groups were as follows: G2 = 0.85 (0.0–49.55) mg/l, G3 = 0.0 (0.0–4.53) mg/l and G4 = 0.0 (0.0–7.5) mg/l); in G1, values of 0.00 (0.0–0.00) mg/l were recorded. SAA concentrations differed significantly among tested groups (*χ*^2^ = 13.56, *df* = 3, *P* = 0.004). In particular, this marker was significantly higher in G2 when compared to G1 controls (*Z* = 3.62, *df* = 1, *P* = 0.002), while no significant differences were detected between G3 or G4* versus* G1. No differences in SAA concentration were detected among G2, G3 and G4 (Fig. 1).

The proportion of cats showing increased alpha 2 and gamma globulins on SPE differed significantly among experimental groups (*χ*^*2*^ = 15.56, *df* = 3, *P* = 0.0014 and *χ*^*2*^ = 22.225, *df* = 3, *P* < 0.0001, respectively) (Fig. 1). In particular, the proportion of cats showing increased alpha 2 globulins on SPE was significantly higher in cats from G2, G3 and G4 (percentage ± SE: G2 = 80.0% ± 8.9, G3 = 70.0% ± 10.2, G4 = 75.0% ± 12.5) compared to healthy controls (G1 = 20.0% ± 10.3 and 0% ± 0) (G1* versus *G2: *χ*^2^ = 13.23, *df* = 1, *P* = 0.0003; G1* versus* G3: *χ*^2^ = 9.05, *df* = 1, *P* = 0.003; G1* versus* G4: *χ*^2^ = 8.59, *df* = 1, *P* = 0.003). A comparable trend was observed in the proportion of cats showing increased gamma globulins on SPE, which was significantly higher in cats from G2, G3 and G4 (G2 = 65.0% ± 10.7, G3 = 50.0% ± 11.2, G4 = 58.3% ± 14.2) compared to healthy controls (G1 = 0% ± 0) (G1* versus* G2: *χ*^2^ = 20.28, *df* = 1, *P* < 0.0001; G1* versus* G3: *χ*^2^ = 14.15, *df* = 1, *P* = 0.0002; G1* versus* G4: *χ*^2^ = 14.60, *df* = 1, *P* = 0.0001). No significant difference in the proportion of animals displaying increased alpha 2 globulins or increased gamma globulins between G2, G3 and G4 was observed (Fig. 1). In all cases, gammopathy was polyclonal (not shown).

## Discussion

In this study, SAA concentration was significantly higher in cats with neoplastic or inflammatory conditions (G2) than in healthy controls (G1), whereas no significant differences in SAA concentrations were noted between *Leishmania*-infected (G3) or -exposed (G4) cats compared to healthy animals. Previous studies had shown that elevated SAA levels are associated with clinically overt cases of feline *H. felis* and *B. vogeli* infection [25]. In contrast, our data suggests that SAA concentration is not reliably linked to FeL.

The percentages of animals showing elevated alpha 2 globulins were significantly higher in infected and exposed cats (G3 and G4) compared to healthy controls (G1), suggesting that the elevation of these markers, indicating an acute phase response, is a significant feature of FeL. However, elevated alpha 2 globulins were also observed in cats with neoplastic and inflammatory conditions compared with healthy controls, and therefore may not be considered robust indicators of FeL. Nevertheless, increased alpha 2 globulins are a recognised feature of infection in dogs [16], whereas previous studies on FeL have revealed either no significant difference in alpha 2 globulin levels between infected and uninfected cats [29], or significantly lower alpha 2 globulins in FeL-infected cats [28]. In both above-mentioned studies [28, 29], the median alpha 2 globulin levels for infected cats remained within the reference range. However, the methodology implemented in our study differed in that we compared the proportion of cats with elevated alpha 2 globulins, rather than the median alpha 2 globulin values between groups, thus aiming to answer the question of whether elevation of alpha 2 globulin values above the reference range is more common among FeL-infected cats compared to non-infected cats.

The results suggest that elevated gamma globulins are a significant feature of *Leishmania* infection in cats, although (as reported for alpha 2 globulins) no significant differences in gamma globulin concentrations were detected  between *Leishmania*-infected or -exposed cats and cats with other neoplastic or inflammatory conditions. Hyperglobulinaemia has been recorded in most clinical cases of FeL [5, 14, 15]. In our study, all cases of gammopathy were polyclonal, in agreement with published typical laboratory abnormalities associated with CanL [16] and the diagnostic recommendations for FeL [29].

There was no significant difference in SAA serum concentration, or in the percentage of animals showing elevated gamma or alpha 2 globulins, between either group of animals infected by or exposed to *L. infantum* (G3 and G4) and animals affected by neoplastic or inflammatory conditions (G2). This result suggests that gamma and alpha 2 globulins are not specific markers of FeL; however, this does not preclude their role as part of a diagnostic plan and in monitoring and assessing disease prognosis . The absence of a significant difference in gamma and alpha 2 globulins between these groups of cats suggests that these markers may be an important feature of both active infection and parasite exposure. It is also possible that G3 cats had a very low parasitaemia that went undetected by qPCR [cf. 34].

A limitation of our study is that the ‘healthy control’ animals (G1) and the neoplastic and inflammatory disease group (G2) were not tested to rule out *L. infantum* infection. However, these cats were from ZVL-free areas of Switzerland (G1) and the UK (G2), respectively, and included laboratory animals bred and kept in a controlled environment as well as blood donor cats. Furthermore, G3 and G4 animals were not tested for concurrent inflammatory disease, such as feline infectious peritonitis, sepsis or gingivitis, all of which would induce an acute phase response and elevated gamma globulins.

In conclusion, the results of this study suggest that elevated alpha 2 and gamma globulins in cats may not be used to differentiate between infection and/or exposure to *L. infantum* and the occurrence of other inflammatory conditions.

## Data Availability

The datasets used and analysed during the current study are available from the corresponding author on reasonable request.

## Abbreviations

APP: Acute phase protein
CRP: C-reactive protein
ELISA: Enzyme-linked immunosorbent assay
FeL: Feline leishmaniosis
IFAT: Immunofluorescence antibody test
qPCR: Quantitative (real-time) PCR
SAA: Serum amyloid A
SPE: Serum protein electrophoresis
ZVL: Zoonotic visceral leishmaniosis
